# Wearable Biosensor Smart Glasses Based on Augmented Reality and Eye Tracking

**DOI:** 10.3390/s24206740

**Published:** 2024-10-20

**Authors:** Lina Gao, Changyuan Wang, Gongpu Wu

**Affiliations:** 1 School of Opto-Electronical Engineering, Xi’an Technological University, Xi’an 710021, China; gaolina@st.xatu.edu.cn (L.G.); wartensie@st.xatu.edu.cn (G.W.); 2 School of Computer Science, Xi’an Technological University, Xi’an 710021, China

**Keywords:** wearable biosensors, augmented reality, eye tracking, smart glasses

## Abstract

With the rapid development of wearable biosensor technology, the combination of head-mounted displays and augmented reality (AR) technology has shown great potential for health monitoring and biomedical diagnosis applications. However, further optimizing its performance and improving data interaction accuracy remain crucial issues that must be addressed. In this study, we develop smart glasses based on augmented reality and eye tracking technology. Through real-time information interaction with the server, the smart glasses realize accurate scene perception and analysis of the user’s intention and combine with mixed-reality display technology to provide dynamic and real-time intelligent interaction services. A multi-level hardware architecture and optimized data processing process are adopted during the research process to enhance the system’s real-time accuracy. Meanwhile, combining the deep learning method with the geometric model significantly improves the system’s ability to perceive user behavior and environmental information in complex environments. The experimental results show that when the distance between the subject and the display is 1 m, the eye tracking accuracy of the smart glasses can reach 1.0° with an error of no more than ±0.1°. This study demonstrates that the effective integration of AR and eye tracking technology dramatically improves the functional performance of smart glasses in multiple scenarios. Future research will further optimize smart glasses’ algorithms and hardware performance, enhance their application potential in daily health monitoring and medical diagnosis, and provide more possibilities for the innovative development of wearable devices in medical and health management.

## 1. Introduction

In recent years, the field of wearable biosensors has made remarkable progress and development [1], and its development has been particularly prominent in sensor technology innovation and device design. Especially in the context of the rapid growth of head-mounted displays, augmented reality (AR) technology in wearable devices, especially the integration of smart glasses, has unique advantages and application potential in several fields of innovation and application prospects.

Particularly in the context of the rapid development of head-mounted displays, wearable computing technologies are becoming more prevalent and are increasingly equipped with more user-friendly interfaces. This trend has facilitated the widespread use of wearable devices in health monitoring [2], motion tracking [3], medical diagnostics [4], and daily life [5]. At the same time, wearable devices’ lightweight and high-performance design makes them easier to integrate into daily life, further enhancing the value of wearable sensors in personal health management and medicine. With the continuous development of science and technology, wearable biosensors will make breakthroughs in more fields and improve people’s lives. Among them, wearable smart glasses with augmented reality (AR) can visually superimpose different external detailed information on the natural environment, allowing the user to interact in both worlds. Many studies have shown that AR will be more favorable in other areas to help people perform various tasks with many positive results [6,7,8].

Integrating line-of-sight tracking technology into augmented reality devices can provide a more natural and immersive user experience and enhance user interaction with virtual reality [9,10,11]. Firstly, this technique enables intuitive interface control based on the user’s gaze, where we can select or activate specific virtual elements or information by looking at them without the need to use our mobile phone’s voice or controllers, making the interaction more natural and efficient [12,13,14,15]. In education and training, users’ attention and behavior can be guided and assessed, and we can display guidance information on a monitor or operator panel. At the same time, the line of sight tracks the user’s direction of gaze to determine the display of the current gaze critical area [16,17,18]. In scenarios such as industrial maintenance [19], surgical operations [20], and driver training [21], such real-time feedback can improve learning and operational safety. View tracking technology can also collect user concerns and interests to provide personalized content and experiences in AR applications. For example, in shopping or museum visits, the system can recommend relevant products or exhibit information based on the user’s eye direction and dwell time, making the user experience more personalized and targeted [22,23,24,25,26]. In multi-user AR environments, line-of-sight tracking technology can enhance the naturalness and collaboration of social interactions [27,28,29]. Users can see the direction of other people’s gazes and understand what they are focusing on or the area they are focusing on to coordinate collaborative tasks better or simulate face-to-face communication in virtual meetings to enhance the efficiency of teamwork.

The hardware used in this study is commercially available and widely used by many researchers in pupil detection and eye tracking. We chose this setup to ensure that our results are comparable to existing research and to focus on the algorithmic aspects of advancing the problem rather than introducing new hardware configurations. In summary, our main contribution is combining a data-driven deep learning approach with a binomial mapping model based on physical laws. Both advantages are fully exploited by obtaining high-precision pupil center position through the deep learning method and then estimating the direction of vision through the physical model. This combination avoids the limitations of a single process and enables the system to maintain high accuracy and robustness in various complex environments. Compared to traditional CNN methods, our innovation is to constrain the pupil boundaries by a geometric model so that the pupil center estimation not only relies on data-driven feature learning but also calibrates with the help of a physical model. As a result, our method can maintain high accuracy even under data scarcity or drastic changes in illumination. In addition, by introducing more training data and data enhancement strategies, the deep learning model has a strong generalization ability to adapt to the differences in eye structures of different users, which enhances the universality of the system.

## 2. Related Work

In recent years, related scholars have researched estimation algorithms applicable to head-mounted eye tracking devices and proposed many fruitful algorithms. Eye tracking can be classified into two main categories, invasive and non-invasive, mainly based on the contact method. Among them, invasive methods involve physical contact, such as installing sensors on the eye’s surface, which can provide higher precision measurements but have obvious limitations regarding comfort and applicability. In contrast, non-invasive methods do not require direct contact with the eye, and gaze estimation is mainly realized through remote monitoring techniques. Thus, they have significant advantages in comfort and acceptability and are especially suitable for head-mounted devices. In terms of detection methods, eye tracking algorithms can be further divided into several types, such as mechanical, current, electromagnetic, and optical video image methods, which are based on different principles and used in various scenarios. In this paper, the wearable astute eye mainly adopts the optical video image method, which involves primarily taking images of the face or eyes through an eye movement camera, utilizing image processing-related algorithms to extract the eye movement features, thus estimating the gaze direction of the human eye.

In a head-mounted eye tracking device, the mapping model for gaze estimation is crucial to determining the accuracy and reliability of the system prediction. Currently, researchers have proposed various models for improving the accuracy and efficiency of gaze estimation. Standard mapping models include geometric models and machine learning models. The geometric model mainly estimates the gaze direction by calculating the relationship between the center of the eyeball and the corneal reflection point based on the geometric properties of the eye structure. This method has high computational accuracy but requires device calibration accuracy. Table 1 summarizes the eye characteristics, main error factors, and other common factors in 2D gaze estimation methods. We can see that traditional geometric methods can theoretically achieve high accuracy, especially in ideal environments, but have many limitations, such as the need for personalized calibration, poor robustness to head motion, and sensitivity to light and camera position.

Machine learning models are used to predict gaze direction by training models with large amounts of labeled data using techniques such as deep learning. Such methods can show strong robustness and good adaptability in multiple complex environments. Convolutional neural network (CNN)-based gaze estimation methods focus on learning a mapping model between ocular appearance features and gaze points by constructing a deep multilayer neural network. This method utilizes the hierarchical nature of CNNs to automatically extract low-level to high-level visual features from the input raw image. It correlates these features with gaze angles through the deep network structure for accurate gaze estimation. CNN-based gaze estimation methods usually require frequent iterative training with multiple optimizations over large-scale datasets, enabling the network to collect the subtle changes between eye appearance and gaze direction. During this continuous training process, the network improves its prediction ability for standard samples and enhances the robustness of gaze estimation for complex scenes or extreme conditions. According to the different learning methods, CNN-based learning models are supervised, weak/semi/self-supervised, and unsupervised. We make a comparative analysis in Table 2. In short, CNN-based gaze estimation methods map the visual information in the original image to the gaze point prediction results through the framework of deep learning and apply different learning strategies under various degrees of supervision to adapt to other data scenarios and application requirements.

## 3. Materials and Methods

### 3.1. Overall Structural Design

**Figure 1:**
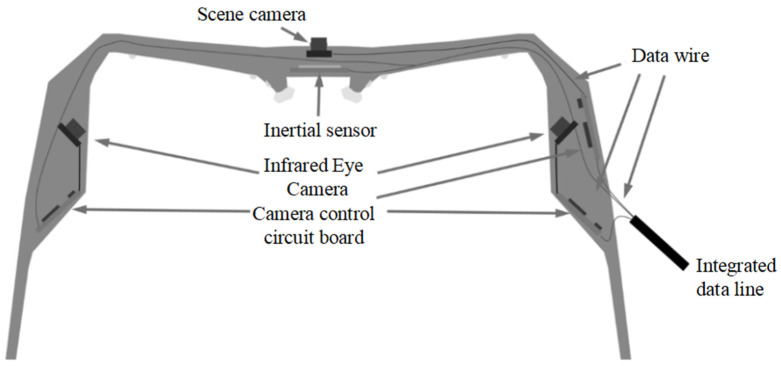
Biological smart glasses structure diagram.

This study uses wearable AR line-of-sight tracking glasses as the hardware platform, integrating the left and right eyeball camera modules with the scene camera module. By capturing eye images, the system realizes the computation of the direction of view and fuses the direction of view and the point of gaze with the scene information. The main body of the AR glasses used is the Huawei Vision Glass, with a built-in 120-inch virtual display and a thin and light design optical module to achieve a slim body of about 21 mm, which is light and comfortable to wear. At the same time, the device supports independent adjustment for myopia up to 500 degrees in one eye and has a pupil distance adaptive function. The eyeball camera module adopts a reflective structure to capture the eyeball area in the near-infrared wavelength band and acquire eyeball images of both eyes. The module has a built-in infrared light source for supplemental light, and the surface of the reflective lens has been coated with a high-transmittance visible infrared reflective coating. The scene camera is located in the center of the main frame of the glasses and configured to capture the scene beyond the wide-angle lens. The scene camera captures information about the real-world scene when the vision-tracking glasses are worn alone. The smart glasses monitor the user’s line of sight to ensure that it is always focused on the gaze area, reducing errors caused by distractions. In addition, the intelligent focusing function based on eye tracking can automatically adjust the display area and support the superimposition of multimodal physiological sensors in the display area, thus providing users with a more personalized and precise operating experience. The eyeglass body integrates the scene camera module and the eye camera module with a lightweight structure that is convenient to wear. The design structure of the physical display is shown in Figure 1.

**Figure 2:**
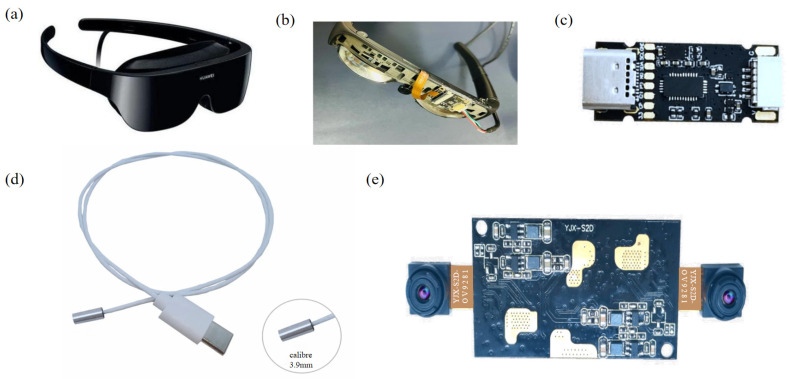
Physical diagram of the bio-smart glasses and the structure of the associated sensors: (**a**) Exterior device diagram of Huawei Vision Glass; (**b**) Diagram of the internal structure of the BioSmart Glass; (**c**) Eye camera sensor decoding board; (**d**) Physical diagram of the endoscope; (**e**) Scene camera sensor.

The physical drawing of the smart glasses is shown in Figure 2a, and the internal structure is shown in Figure 2b. The eye camera sensor is the critical component in this project, and it is responsible for collecting the user’s eye movement information in real time. A miniature high-resolution camera fitted in front of each eye can capture the subtle movements of the eyeballs. The camera sensor employs an infrared light source and a near-infrared filter to reduce ambient light interference and provide more accurate eye tracking data. Its sampling frequency is up to 300 Hz to ensure that every detail of the eye movement is collected. Figure 2c shows the decoding board of the eyeball camera, with a built-in image processor and digital signal processor, outputting high-quality image quality with a maximum pixel of 1280*720 that can transmit up to 30 fs and deficient power consumption and very low signal delay. The endoscope adopted Sensor: OV9734 1/9 inch, Pixel: 1280 × 720, Frame rate: 30 fps, Field of view: 88°, Depth of Field: 15–100 mm, Output format: MIPI signal output, Module size: Diameter: 3.9 mm*15 mm, as shown in Figure 2d. The scene camera is used to collect the scene image in the direction of the user’s line of sight, mounted on the front of the glasses, with a high-resolution wide-angle lens that can cover the user’s entire field of view, as shown in Figure 2e. A high-resolution scene camera is selected to capture scene images in the direction of gaze, with a resolution of 1080 p or higher and a frame rate of 30 fps or higher.

### 3.2. Introduction to the Dataset

In the pupil recognition study, the video-based dataset LPW and the image-based dataset Excuse are used to fully use the advantages of the two types of data and improve the model’s generalization ability. With video data, the trajectory of pupil changes at different time points can be collected, which helps to study the real-time tracking of the pupil and the change of posture; video data can provide continuous pupil position, which allows the model to maintain stable recognition performance even when there is noise or transient inaccuracy; in real applications, the user’s head or eyeballs may constantly move, and the video dataset can help to validate the system’s performance in dynamic scenes. The image-based dataset, on the other hand, contains single-frame still images, which can help the model accurately recognize the edges and positions of the pupils, especially in the training phase of the model, as still images usually have higher resolution. Although the LPW dataset is more in line with the research goal of real-time tracking, the manually labeled images of the Excuse dataset play an essential role in the model’s accuracy enhancement and the reliability of pupil detection. By combining these two types of datasets, the model can perform well in both dynamic and static scenarios, is suitable for both scenarios requiring real-time pupil tracking, and can achieve high precision pupil detection. 

**Figure 3:**
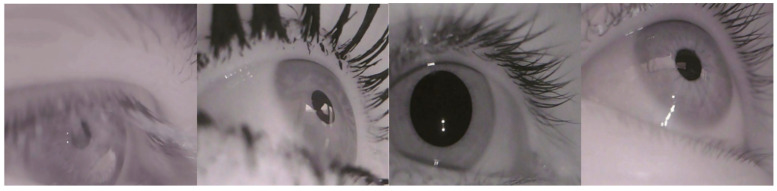
Selected images of the LPW dataset.

**Figure 4:**
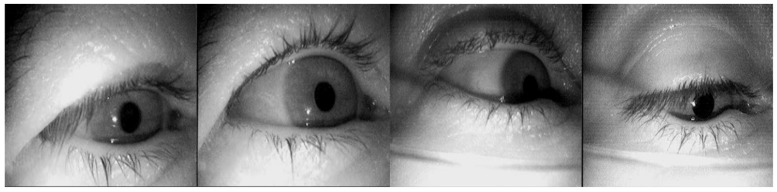
Selected images of the Excuse dataset.

Specifically, the pupil detection dataset LPW [56] was data collected by 22 participants using a state-of-the-art dark pupil head-mounted eye tracker. As shown in Figure 3. Each video in the dataset consists of approximately 2000 frames with a resolution of 640 × 480 pixels, recorded at approximately 95 FPS, for a total of 130,856 video frames. The Excuse [57] dataset was recorded using a head-mounted system driving on the road to capture nine datasets, with the remaining eight datasets captured during a search task completed in a supermarket. As shown in Figure 4. This dataset contains a total of 38,000 manually labeled eye images. We divided the two datasets into 70% for training, 15% for validation, and 15% for testing. These images are highly challenging because lighting conditions often change rapidly. In addition, reflections occur on spectacles and contact lenses. These data reflect the results of standard eye tracking experiments outside the laboratory and document other studies that do not focus on pupil detection. Both datasets cover a variety of indoor and outdoor scenarios, including shadows, reflections, light changes, mascara, eyeglasses, eyelashes, and highly off-axis pupils. The dataset does not contain predefined categories. Instead, the annotations provide precise pupil positions and shapes used as target values for regression and detection tasks.

### 3.3. Eye-Pupil-Based Tracking Recognition

Eye tracking technology based on deep learning algorithms can achieve high precision pupil recognition in smart glasses, which provides essential support for subsequent eye posture measurement and vision direction calculation. Therefore, to address the problems of insufficient individual-specific accuracy, lack of robustness, and inability to run in the real-time existing eye segmentation methods, we implemented eye gaze tracking based on the UNet network. The technique achieves a tracking frequency of over 100 Hz, enabling real-time line-of-sight tracking. Combined with the display function of AR glasses, it can further realize efficient human-computer interaction and collaboration in the visual range. Through deep learning algorithms, we process and analyze the left and right eye images collected by the eye camera to accurately identify the pupil region in the picture. The binocular vision system focuses on the tracking and recognition of the eye pupil, which achieves accurate recognition and measurement of the eye pupil region by processing and analyzing the eye image, combining pattern recognition and deep learning algorithms, extracting the pupil features, obtaining the current location of the pupil, and then achieving the accurate measurement of the two-axis rotational attitude of the eye and the pupil diameter. Eye gaze tracking based on unit networks, while full convolutional network models perform well on many datasets, they come at the cost of computational complexity, limiting their feasibility in real-time applications. In contrast to the improved model size of only 1.2 MB, the domain-specific enhancement scheme in the model helps to generalize across a variety of challenging conditions, while the boundary-aware loss function and loss scheduling strategy train the deep semantic segmentation model, which helps in producing coherent regions with clear region boundaries.

**Figure 5:**
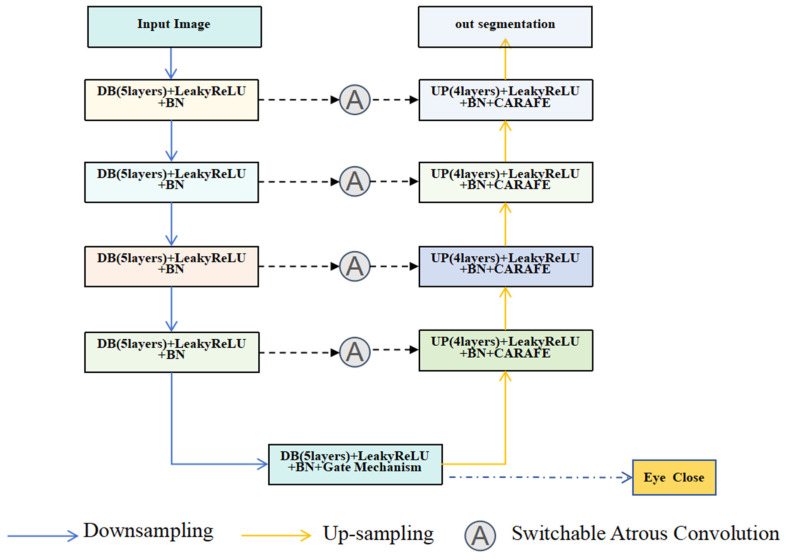
Structure of the pupil detection network. DB refers to down block, UP refers to upb lock, and BN stands for batch normalization.

The model structure is shown in Figure 5, and the whole network consists of five down blocks and four up blocks. DB refers to down block, UP refers to upb lock, and BN stands for batch normalization. These modules perform downsampling and upsampling operations on the input feature maps, respectively. Each down block consists of four convolutional layers, which use LeakyReLU as the activation function, which helps to alleviate the ‘dead neuron’ problem, thus improving the stability and expressiveness of the model. We connect a 2 × 2 average pooling layer behind each downsampling block, whose primary function is to reduce the size of the feature map while retaining important feature information. We call the last down block the Bottleneck Layer, whose primary role is to compress the feature information of the whole model into a small tensor, whose size is 1/16 of the input resolution. The tensor, though small in size, contains the global information of the whole input image. We also perform eye closure detection at the bottleneck layer, which significantly impacts the final pupil segmentation results, as the accuracy of the pupil segmentation task is highly dependent on whether the eyes are open or not. When the eyes are closed, the pupil is not visible, which will make the pupil segmentation model produce incorrect predictions or invalid results with such inputs, so we use the key features included to determine whether the eyes are closed or not to output the results of the eye closure detection.

In the Up-Block, each up-sampling module consists of four convolutional layers, which also use the LeakyReLU activation function, which allows the feature maps generated during the up-sampling process to maintain good non-linear representations and helps to reconstruct the detail information at high resolution. Content-aware reassembly (CARAFE) is also used in the decoder, where features are subsequently up-sampled by the CARAFE layer to enable accurate feature reassembly. In addition, all the upsampling modules receive additional information from their corresponding downsampling modules via Skip Connection. This Skip Connection is an effective strategy that allows the upsampling modules to directly access the feature representations with different spatial granularities extracted from the earlier downsampling modules, thus enhancing the feature reconstruction capability of the model. This design not only improves the training efficiency of the network but also preserves more detailed information in the input image, which ultimately enhances the overall performance capability of the model.

Specifically, in the case of eye closure detection, the up-sampling process can be selectively switched off by a gating mechanism, thus avoiding ineffective pupil segmentation operations. This mechanism decides whether to turn on the up-sampling branch based on the probability value of the eye closure detection. The specific steps are to control a gating variable G before the up-sampling starts using the output $P_{close}$ eye closure probability from the eye closure detection module. The gating variable *G* can be given by:

(1) $$G=Heavside(1-P_{close}-\varepsilon )$$

Here, ε is a very small threshold that ensures that the gating is triggered only when close to eye closure. Heavside function is used to convert the probability to a 0 or 1 control signal.

We use the CARAFE layer for up-sampling, and its core idea is to use the content of the input features to guide the model up blocks, to achieve more accurate and efficient feature reassembly. However, traditional up-sampling methods often use bilinear interpolation or inverse convolution, which cannot fully take into account the content of the input features and may lead to the loss of details during the up-sampling process, especially the edge regions of the object. The CARAFE is improved by the following steps: the first step is to predict the reorganization kernel of each target location based on its content, and the second step is to reorganize the features with the predicted kernel. Assume that given a feature map A of size C×H×W, for each target location q=(u,v) in the output feature map, there is a corresponding source location p=(s,t) in the input feature map. It can be expressed as:

(2) $$s=\left\lfloor u/r\right\rfloor ,t=\left\lfloor v/r\right\rfloor$$

The kernel prediction formula becomes:

(3) $$K_{q}=\theta (B(A_{p},m))$$

Here, $B(A_{P},m)$ is the m×m sub-region of A centered on p.

The expression for the characteristic reorganization is:

(4) $${A_{q}}^{\prime }=\omega (B(A_{P},m),K_{q})$$

Here, ${A_{q}}^{\prime }$ in feature recombination denotes that the value of the $A^{\prime }$ position in the output feature map is q. It is obtained by the content-aware recombination module ω by combining or convolving the neighborhood $B(A_{P},m)$ of the $A_{p}$ with the kernel $K_{q}$.

During model training, we use the joint loss function to optimize the whole model by combining the eye closure detection loss and pupil segmentation loss. The specific expression is:

(5) $$l_{total}=\lambda_{1}l_{pupil}+\lambda_{2}l_{eye\_closure}$$

Here, $l_{pupil}$ is the loss function of pupil segmentation, and we use the Dice loss for measuring the accuracy of pupil segmentation. It can be expressed as:

(6) $$L_{pupil}=1-\frac{2\sum_{i=1}^{N}(y_{i}*{\hat{y}}_{i})}{\sum_{i=1}^{N}\left(y_{i}+{\hat{y}}_{i}\right)}$$

Here, $y_{i}$ and ${\hat{y}}_{i}$ are the real labels and model-predicted pupil segmentation results, and N is the total number of pixels in the image.

For the loss function of closed-eye detection, we use Binary Cross-Entropy Loss (BCEL) for binary classification. The cross-entropy loss is robust and defined as:

(7) $$L_{eye\_closure}=-\frac{1}{N}\sum_{i=1}^{N}\left(y_{i}\log{\hat{y}}_{i}+\left(1-y_{i}\right)\log\left(1-{\hat{y}}_{i}\right)\right)$$

Here, $y_{i}$ is the true label for closed-eye detection (1 for closed eyes and 0 for open eyes), ${\hat{y}}_{i}$ is the probability value predicted by the model, and N is the total number of pixels in the image.

### 3.4. Calculation of Gaze Direction and Gaze Drop Combined with Scene Information

There are four standard mapping models for vision estimation: first-order polynomial regression models, second-order polynomial regression models (including simplified second-order polynomial regression models), Gaussian process regression models, and artificial neural network regression models. We performed a comparative analysis in Table 3. Considering that the number of calibrations should be sufficiently small and simple for the actual use of head-mounted coulometers, artificial neural networks, a model that requires a large amount of calibration data for the training of the network parameters, are also not applicable in the estimation of head-mounted coulometers. We propose to use a second-order polynomial regression model to calculate the gaze fall point. Fusion of gaze direction and gaze fall point combined with scene information: a calibration algorithm is established to fuse the gaze direction and gaze fall point calculated from the left and right eye images with the scene information obtained from the AR eyecup scene display images to output the subject’s binocular gaze direction and gaze fall point in the scene.

**Figure 6:**
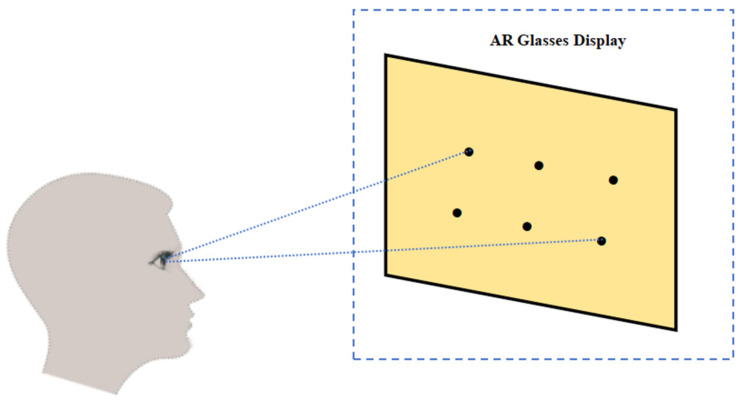
Demonstration diagram of smart glasses sight calibration.

The design of the calibration system in the AR scene is mainly divided into two parts: the first is the calibration part, which is completed by setting six particular calibration points on the scene graph and solving the corresponding pupil center coordinates to establish the mapping equations, thus completing the calibration of eye movements. In the calibration process, we first let the subjects keep their heads still and observe the corresponding calibration points with the system’s cue tone, where the corresponding scene calibration points are shown in Figure 6 below. At this time, the software system noted down the pupil center coordinates when the eyes were looking at these six points and extracted the coordinate values of these six calibration points simultaneously. The expression of this mapping relationship is:

(8) $$\left\{\begin{array}{l} u=A_{0}+A_{1}x+A_{2}y+A_{3}x^{2}+A_{4}xy+A_{5}y^{2}\\ v=B_{0}+B_{1}x+B_{2}y+B_{3}x^{2}+B_{4}xy+B_{5}y^{2} \end{array}\right.$$

Here, (u,v) is the coordinates of a predefined standard point in the scene, (x,y) is the pupil vector corresponding to the standard shop, A and B are the coefficients of the mapping polynomial with the specific expression:

(9) $$\begin{array}{l} \left\{\begin{array}{l} A={\left.\left\{A_{0}\right.A_{1}A_{2}\ldots A_{m-1}\right\}}^{T}\\ B={\left.\left\{B_{0}\right.B_{1}B_{2}\ldots B_{m-1}\right\}}^{T} \end{array}\right.\\ m=1+\sum_{i=1}^{n}\left(1+i\right) \end{array}$$

Second the gaze point display part, when the eyes gaze at the scene, the real-time collected pupil vector is substituted into the mapping polynomial to find out the actual gaze point of the eyes and display it on the scene. The specific expression is:

(10) $$\left\{\begin{array}{l} A=M^{-1}u\\ B=M^{-1}v \end{array}\right.$$

According to the relevant properties of mathematical equations, it can be seen that the matrix M is only invertible to minimize the mean square deviation between the calibration points (u,v) in the scene and the estimated coordinate values (x,y) derived through the mapping polynomial. The mapping polynomial can be obtained by substituting the derived A and B into Equation (8), and the obtained pupil vectors can be substituted into this polynomial to find out the coordinates of the gaze point of the human eye in the scene at this time.

## 4. Results

### 4.1. Analysis of Pupil Recognition Effect

**Figure 7:**
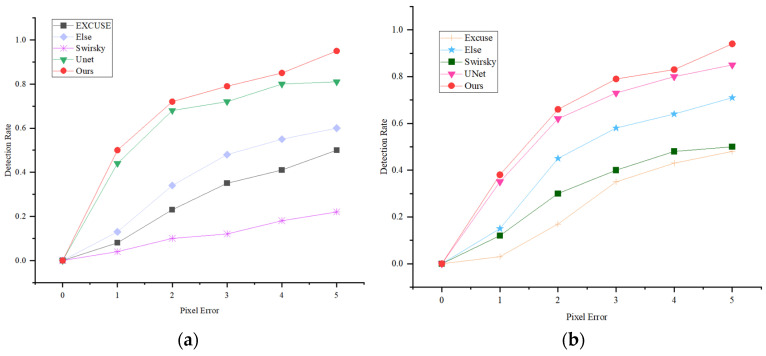
Figure shows the results of the comparative analyses of the models. (**a**) Results of analyses in dataset LPW; (**b**) Results of the analyses in the dataset Excuse.

**Figure 8:**
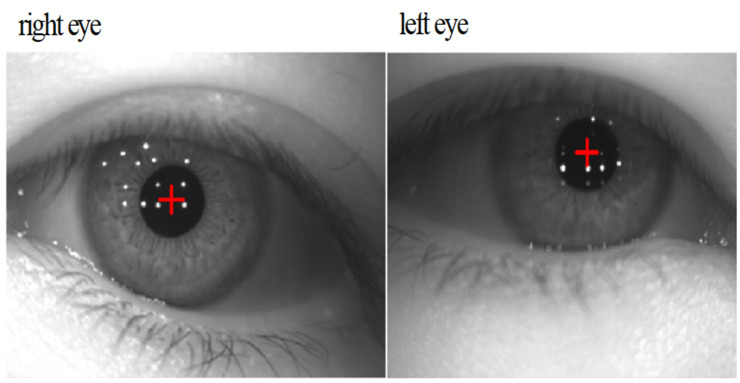
Pupil recognition display results.

In this paper, the experimental environment is Pytorch 1.8.1, and the pupil recognition model was trained using the Adam optimizer, with an initial learning rate of 0.001 for training, 0.9 for the first decay rate, 0.999 for the second decay rate, and a batch size of eight. A common way of looking at the performance of pupil detection algorithms is to perform an error rate of 5 pixels; if the Euclidean distance between the center of the detected pupil and the center of the ground truth pupil is less than 5 pixels, the detection is considered correct. Otherwise, the detection is assumed to be incorrect. To evaluate the state-of-the-art of the proposed pupil detection model, the benchmark model Unet and the pupil detection algorithms ElSe, ExCuSe, and Swirsky are compared for pupil detection performance under the same experimental environment and experimental dataset. The detection results of the pupil center within the 5-pixel error of the compared models are shown in Figure 7, and it can be seen that the model in this paper predicts the pupil center with a smaller error than all the other models in the comparison, and the specific detection results are shown in Figure 8. Through the analysis, it is evident that the detection structure of the LPW dataset outperforms the EXCUSE dataset when considering the same pixel error threshold. This suggests that the LPW dataset provides more robust and accurate results for gaze estimation tasks or similar applications, highlighting its superior suitability for achieving precise measurements under consistent conditions.

### 4.2. Line-of-Sight Estimation Evaluation Indicators and Results

Accuracy Assessment of Line-of-Sight Estimation Systems Accuracy refers to the ability of a system to predict or reproduce relative gaze positions reliably, and the assessment process involves quantifying the accuracy of the system’s predictions, which is usually accomplished through different statistical metrics. One commonly used evaluation metric in classification problems is accuracy, which measures the proportion of all predictions that the system correctly predicts, especially in dichotomous or multiclassification tasks. A confusion matrix analyzes the system’s performance in classification tasks by counting the number of correct and incorrect predictions made. Accuracy can be calculated using the following formula:

(11) $$m_{precision}=\frac{\left(TP_{b1}+TP_{b2}+\ldots +TP_{bn}\right)}{\left((TP_{b1}+TP_{b2}+\ldots +TP_{bn})+(FP_{b1}+FP_{b2}+\ldots +FP_{bn})\right)}$$

Here, TP is the true positive prediction, and FP is the false positive prediction for consecutive samples $b_{1}$-$b_{n}$.

For the regression problem of continuous value prediction, root mean square error (RMSE) is one of the commonly used metrics. RMSE is used to measure the deviation of the system’s prediction results from the actual values. It is the square root of the mean of the sum of the squares of all the prediction errors of the system, and a smaller RMSE indicates a higher accuracy of the system’s prediction. The specific expression is shown as follows:

(12) $$RMSE=\sqrt{\frac{1}{n}\sum_{i=1}^{n}{\left(b_{i}-a_{i}\right)}^{2}}$$

Here, $a_{i}$ denotes the actual gaze position (true value), $b_{i}$ denotes the gaze position estimated by the system, and *n* is the total number of samples.

Angular resolution is a specialized metric for line-of-sight estimation systems that describes the deviation between the system’s estimated angle of gaze and the actual angle of gaze. Angular resolution is usually expressed in degrees (°); the smaller the deviation, the more accurate the system is, and measures the average error between the estimated gaze angle α and the true gaze angle. The specific formula is:

(13) $$\alpha =\arccos\left(\frac{a•b}{\left|a||b\right|}\right)$$

**Figure 9:**
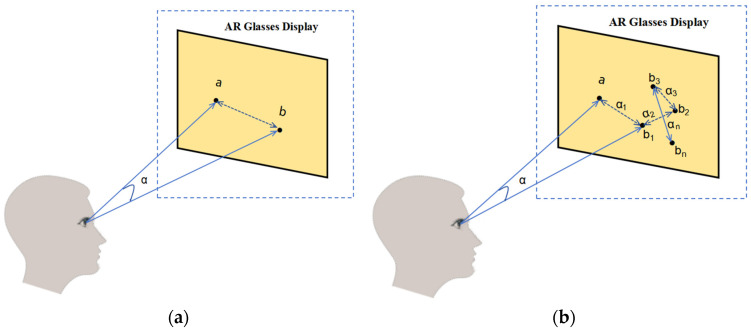
Evaluation methods for line-of-sight estimation system: (**a**) Error between actual gaze position a, and estimated gaze position b; (**b**) Error between actual gaze position a and continuous gaze $b_{1}$–$b_{n}$.

As depicted in Figure 9a, the angular deviation α between the system’s estimated gaze point (b) and the actual gaze point (a) is illustrated in a simple scenario. This figure is a foundational diagram for evaluating angular resolution, facilitating researchers’ understanding of the system’s error in estimating a single gaze point. A smaller angle α indicates that the system’s estimated gaze point is closer to the actual gaze point, reflecting higher prediction accuracy. Thus, angular resolution is crucial for assessing the system’s accuracy, particularly in single-point scenarios.

Figure 9b illustrates the relationship between multiple gaze points (a1, a2, …, an) and the corresponding system-estimated positions (b1, b2, …, bn). This diagram aids researchers in comprehending the overall prediction accuracy of the system in more complex scenarios. If the system accurately predicts all gaze points, the estimated points (b) will closely align with the actual points (a), indicating a higher number of true positive predictions (TP). Conversely, if the system mispredicts certain gaze points, the estimated points (b) will substantially deviate from the actual points (a), resulting in increased false positives (FP) and false negatives (FN).

Using these predictions, we can compute the system’s overall accuracy from the confusion matrix. Figure 9b shows multiple pairs of points, visualizing how the system performs in different sample sets. The higher the accuracy, the closer the actual point (a) is to the estimated point (b), or the smaller the distance between them. In addition, Figure 9b allows for a regression analysis. In this case, b1 to bn represents the multiple predicted points of the system, while a1 corresponds to the actual gaze points. The root mean square error (RMSE) allows quantifying the overall deviation between the predicted (b) and actual (a) points, with smaller RMSE values indicating higher prediction accuracy.

**Figure 10:**
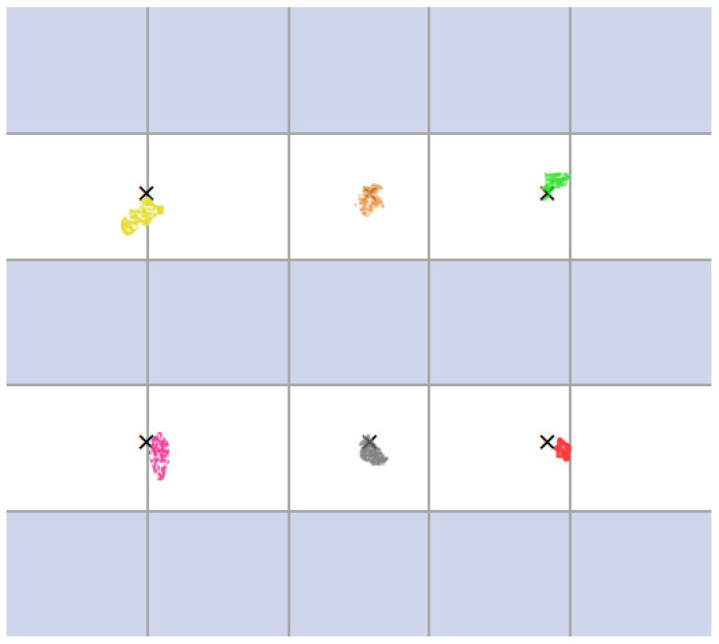
Line-of-sight estimation accuracy results.

In evaluating the line-of-sight estimation performance of the AR glasses, we set up six different target points on the display, as shown in Figure 10. Subjects wore AR glasses and gazed at these predetermined locations, and the system’s estimation error of the subject’s gaze direction was recorded. Here, the 2D coordinates $\left(x^{\prime },y^{\prime }\right)$ of the gaze points estimated by the model are compared with the coordinate values $\left(x,y\right)$ of the preset target points, and the Euclidean distance between the two is calculated as the localization error of the gaze point estimation as follows:

(14) $$E\mathrm{rr}or=\sqrt{{\left(x-x^{\prime }\right)}^{2}+{\left(y-y^{\prime }\right)}^{2}}$$

We set the subject’s distance from the monitor to 1 m to evaluate the system’s accuracy. The overall performance metrics of the system were derived by calculating the gaze estimation at each target position. In real-world testing, we found that although the Gaussian process regression model has a more vital nonlinear fitting ability, it is more data-dependent, and usually, accurate Gaussian process regression requires a large amount of calibrated data for training to obtain a better prediction performance. This increases the user burden in applications such as head-mounted eye tracking devices, as the calibration process can become tedious and time-consuming. At the same time, if the calibration data is small or the data distribution is not uniform, the overfitting problem is prone to occur, especially in noisy data. Therefore, second-order polynomial regression tends to be a more robust choice for tasks that require less calibration and strong generalization. Here, we avoid overfitting or bias due to specific dataset partitioning through N-fold cross-validation. Comparing the performance of the first-order polynomial regression model and the second-order polynomial regression model, their results are analyzed as shown in Table 4.

## 5. Conclusions

We summarize the innovations and application prospects of wearable biosensors, particularly the smart glasses that combine AR technology and line-of-sight tracking. AR smart glasses achieve natural user interaction by superimposing virtual information on real environments, demonstrating their unique advantages and application potential in various scenarios, including education, healthcare, industry, and social networking. Meanwhile, by combining line-of-sight tracking technology, they provide a more innovative and personalized user experience and improve the safety and efficiency of user operations. In terms of hardware, the integrated infrared sensors and microdisplays can track eye movement data with high precision; in terms of algorithms, using the Unet model-based pupil segmentation method improves the accuracy of pupil center estimation. These innovations enable AR smart glasses to have broader application prospects in multiple fields and provide a new direction for the future development of human-computer interaction technology.

## Figures and Tables

**Table 1:** Characteristics of common 2D gaze estimation methods.

Methods	Cameras	Eye Feature	Main Error Factors	References
PCT/ICT-based	1	Pupil/iris center, corner point	Sensitive to head movement, requires looking at multiple calibration points, less practical in moving applications	[30,31,32]
PCRT/ICRT-based	1	Pupil/iris center, glints	Dependent on the detection of the corneal reflection point (glint), with a single light source system, the accuracy decreases significantly when the head deviates from the calibrated position	[33,34,35,36]
	≥2	Pupil/iris center, glints	Multi-point calibration to compensate for kappa angle (deviation between visual and optical axes)	[37]
CR-based	≥1	Pupil center, glints	The accuracy performance during head movement is not as good as in the case of a fixed head, especially when multiple light sources are not coplanar, and the error increases further	[38,39,40]
HN-based	≥1	Pupil center, glints	It requires the light sources to be set at the four corners of the screen and mapped by a single reactivity matrix, thus placing high demands on the accuracy of the light source positions	[41,42,43]

**Table 2:** Comparative analysis of appearance-based attention estimation methods.

Methods	Algorithms	Main Error Factors	References
Conventional machine learning-based	KNN, RF, GP, SVR, ALR	Individual, head motion Head motion, environment	[44,45,46]
CNN-based	Supervised learning model	Supervised CNNs need well-designed architecture and parameters but require many samples and long training. Synthetic images differ from real ones	[47,48,49]
	Weakly/semi/self-supervised learning model:	Performance still relies on having high-quality labeled data and falls behind fully supervised methods	[50,51,52]
	Unsupervised learning model:	It is prone to deviate from the ground truth due to issues such as individual differences.	[53,54,55]

**Table 3:** Comparative analysis of common mapping models for line-of-sight estimation.

Model Type	Model Complexity	Training Difficulty	Generalization Ability	Computation Speed	Advantages	Disadvantages
First-order Polynomial Regression Mode	Low	Low	Moderate	Fast	Simple and efficient and suitable for linear problems	Cannot handle complex nonlinear relationships
Second-order Polynomial Regression Mode	Moderate	Moderate	High	Fast	Can handle some nonlinear relationships, low model complexity, and high computational efficiency	Limited performance with high-dimensional data and limited ability to handle complex nonlinearity
Gaussian Process Regression Model	High	High	Very High	Slow	Excellent generalization ability and handles uncertainty well	High computational complexity, slow training, and inference, especially on large datasets
Artificial Neural Network Regression Model	Very High	Very High	Very High	Moderate	Strong ability to handle nonlinear data and suitable for complex datasets and multi-dimensional inputs	Requires large datasets, prone to overfitting, long training time, and difficult to interpret

**Table 4:** Analysis of line-of-sight estimation accuracy results.

Metric	One-Stage Model	Two-Stage Model	One-Stage Model (Standard Deviation)	Two-Stage Model (Standard Deviation)
Accuracy	85%	92%	±2%	±1%
Recall	0.80	0.90	±0.04	±0.02
F1 Score	0.79	0.89	±0.03	±2
RMSE	1.5°	1.0°	±0.2°	±0.1°

## Data Availability

Data are contained within the article.

## References

[1] Kim D., Choi Y. (2021). Applications of Smart Glasses in Applied Sciences: A Systematic Review. Appl. Sci..

[2] Baashar Y., Alkawsi G., Wan Ahmad W.N., Alomari M.A., Alhussian H., Tiong S.K. (2023). Towards wearable augmented reality in healthcare: A comparative survey and analysis of head-mounted displays. Int. J. Environ. Res. Public Health.

[3] Ham J., Hong J., Jang Y., Ko S.H., Woo W. Smart wristband: Touch-and-motion–tracking wearable 3D input device for smart glasses. Proceedings of the Distributed, Ambient, and Pervasive Interactions: Second International Conference.

[4] Condino S., Montemurro N., Cattari N., D’Amato R., Thomale U., Ferrari V., Cutolo F. (2021). Evaluation of a wearable AR platform for guiding complex craniotomies in neurosurgery. Ann. Biomed. Eng..

[5] Yutong Q., Hang J., Chen J.R., Ng P.J. (2021). The impact of smart glasses on a new generation of users. Int. J. Biomed. Sci. Appl..

[6] Koutromanos G., Kazakou G. (2023). Augmented reality smart glasses use and acceptance: A literature review. Comp. Exp. Res..

[7] Suh A., Prophet J. (2018). The state of immersive technology research: A literature analysis. Comput. Hum. Behav..

[8] Dewan M.H., Godina R., Chowdhury M.R.K., Noor C.W.M., Wan Nik W.M.N., Man M. (2023). Immersive and non-immersive simulators for education and training in the maritime domain—A Review. J. Mar. Sci. Eng..

[9] Chadalavada R.T., Andreasson H., Schindler M., Palm R., Lilienthal A.J. (2020). Bi-directional navigation intent communication using spatial augmented reality and eye-tracking glasses for improved safety in human–robot interaction. Robot. Cim-Int. Manuf..

[10] Yan Z., Wu Y., Shan Y., Chen W., Li X. (2022). A dataset of eye gaze images for calibration-free eye tracking augmented reality headset. Sci. Data..

[11] Plopski A., Hirzle T., Norouzi N., Qian L., Bruder G., Langlotz T. (2022). The eye in extended reality: A survey on gaze interaction and eye tracking in head-worn extended reality. ACM Comput. Surv..

[12] Ribo M., Lang P., Ganster H., Brandner M., Stock C., Pinz A. (2002). Hybrid tracking for outdoor augmented reality applications. IEEE Comput. Graph. Appl..

[13] Rigas I., Raffle H., Komogortsev O.V. (2017). Hybrid ps-v technique: A novel sensor fusion approach for fast mobile eye-tracking with sensor-shift aware correction. IEEE Sens. J..

[14] Syed T.A., Siddiqui M.S., Abdullah H.B., Jan S., Namoun A., Alzahrani A., Nadeem A., Alkhodre A.B. (2022). In-depth review of augmented reality: Tracking technologies, development tools, AR displays, collaborative AR, and security concerns. Sensors..

[15] Kim S.K., Kang S.J., Choi Y.J., Choi M.H., Hong M. (2017). Augmented-reality survey: From concept to application. KSII Trans. Internet Inf. Syst..

[16] Wang Y., Lu S., Harter D. (2021). Multi-sensor eye-tracking systems and tools for capturing student attention and understanding engagement in learning: A review. IEEE Sens. J..

[17] Nixon N., Thomas P.B., Jones P.R. (2023). Feasibility study of an automated Strabismus screening test using augmented reality and eye-tracking (STARE). Eye.

[18] Yu C.Y., Kim J.H., Mostowfi S., Wang F., Oprean D., Seo K. Developing an augmented reality-based interactive learning system with real-time location and motion tracking. Proceedings of the International Conference on Human-Computer Interaction.

[19] Wu S., Hou L., Chen H., Zhang G. Measuring the impact of Augmented Reality warning systems on onsite construction workers using object detection and eye-tracking. Proceedings of the 28th EG-ICE International Workshop on Intelligent Computing in Engineering.

[20] Lu S., Sanchez Perdomo Y.P., Jiang X., Zheng B. (2020). Integrating eye-tracking to augmented reality system for surgical training. J. Med. Syst..

[21] Shahid M., Nawaz T., Habib H.A. (2013). Eye-gaze and augmented reality framework for driver assistance. Life Sci..

[22] Pierdicca R., Paolanti M., Naspetti S., Mandolesi S., Zanoli R., Frontoni E. (2018). User-centered predictive model for improving cultural heritage augmented reality applications: An HMM-based approach for eye-tracking data. J. Imaging.

[23] Meißner M., Pfeiffer J., Pfeiffer T., Oppewal H. (2019). Combining virtual reality and mobile eye tracking to provide a naturalistic experimental environment for shopper research. J. Bus. Res..

[24] Damala A., Schuchert T., Rodriguez I., Moragues J., Gilleade K., Stojanovic N. (2013). Exploring the affective museum visiting experience: Adaptive augmented reality (A2R) and cultural heritage. Int. J. Hum. Dev. Educ..

[25] Jang C., Bang K., Moon S., Kim J., Lee S., Lee B. (2017). Retinal 3D: Augmented reality near-eye display via pupil-tracked light field projection on retina. ACM Trans. Graph..

[26] Lo Valvo A., Croce D., Garlisi D., Giuliano F., Giarré L., Tinnirello I. (2021). A navigation and augmented reality system for visually impaired people. Sensors..

[27] Cai X., Yang Z., Dong L., Ma Q., Miao X., Liu Z. (2023). Multi-user mobile augmented reality with ID-Aware visual interaction. ACM T. Sens. Netw..

[28] Tsamis G., Chantziaras G., Giakoumis D., Kostavelis I., Kargakos A., Tsakiris A., Tzovaras D. Intuitive and safe interaction in multi-user human robot collaboration environments through augmented reality displays. Proceedings of the 30th IEEE International Conference on Robot & Human Interactive Communication.

[29] McGill M., Gugenheimer J., Freeman E. A quest for co-located mixed reality: Aligning and assessing SLAM tracking for same-space multi-user experiences. Proceedings of the 26th ACM Symposium on Virtual Reality Software and Technology.

[30] George A., Routray A. (2016). Fast and accurate algorithm for eye localisation for gaze tracking in low-resolution images. IET Comput. Vis..

[31] Yu M.X., Lin Y.Z., Tang X.Y., Xu J., Schmidt D., Wang X.Z., Guo Y. (2015). An easy iris center detection method for eye gaze tracking system. J. Eye Mov. Res..

[32] Cai H., Yu H., Zhou X., Liu H. (2016). Robust gaze estimation via normalized iris center-eye corner vector. International Conference on Intelligent Robotics and Applications.

[33] Wang J., Zhang G., Shi J. (2016). 2D Gaze Estimation Based on Pupil-Glint Vector Using an Artificial Neural Network. Appl. Sci..

[34] Mestre C., Gautier J., Pujol J. (2018). Robust Eye Tracking Based on Multiple Corneal Reflections for Clinical Applications. J. Biomed. Opt..

[35] Sesma-Sanchez L., Villanueva A., Cabeza R. (2012). Gaze Estimation Interpolation Methods Based on Binocular Data. IEEE Trans. Biomed. Eng..

[36] Zhang T.N., Bai J.J., Meng C.N., Chang S.J. (2012). Eye-Gaze Tracking Based on One Camera and Two Light Sources. J. Optoelectron. Laser..

[37] Zhu Z., Ji Q. (2008). Novel Eye Gaze Tracking Techniques Under Natural Head Movement. IEEE Trans. Biomed. Eng..

[38] Arar N.M., Gao H., Thiran J.P. (2017). A Regression-Based User Calibration Framework for Real-Time Gaze Estimation. IEEE Trans. Circuits Syst. Video Technol..

[39] Sasaki M., Nagamatsu T., Takemura K. Cross-Ratio Based Gaze Estimation for Multiple Displays Using a Polarization Camera. Proceedings of the 32nd Annual ACM Symposium on User Interface Software and Technology.

[40] Sasaki M., Nagamatsu T., Takemura K. Screen Corner Detection Using Polarization Camera for Cross-Ratio Based Gaze Estimation. Proceedings of the 11th ACM Symposium on Eye Tracking Research & Applications.

[41] Hansen D.W., Agustin J.S., Villanueva A. Homography Normalization for Robust Gaze Estimation in Uncalibrated Setups. Proceedings of the 2010 Symposium on Eye-Tracking Research & Applications.

[42] Morimoto C.H., Coutinho F.L., Dan W.H. (2020). Screen-Light Decomposition Framework for Point-of-Gaze Estimation Using a Single Uncalibrated Camera and Multiple Light Sources. J. Math. Imaging Vis..

[43] Choi K.A., Ma C., Ko S.J. (2014). Improving the Usability of Remote Eye Gaze Tracking for Human-Device Interaction. IEEE Trans. Consum. Electron..

[44] Lu F., Sugano Y., Okabe T., Sato Y. (2014). Adaptive linear regression for appearance-based gaze estimation. IEEE Trans. Pattern Anal. Mach. Intell..

[45] Wang Y., Zhao T., Ding X., Peng J., Bian J., Fu X. (2018). Learning a gaze estimator with neighbor selection from large-scale synthetic eye images. Knowl. -Based Syst..

[46] Kacete A., Séguier R., Collobert M., Royan J. (2016). Unconstrained gaze estimation using random forest regression voting. Proceedings of the Asian Conference on Computer Vision.

[47] Zhuang Y., Zhang Y., Zhao H. Appearance-based gaze estimation using separable convolution neural networks. Proceedings of the 2021 IEEE 5th Advanced Information Technology, Electronic and Automation Control Conference (IAEAC), IEEE.

[48] Deng H., Zhu W. Monocular free-head 3d gaze tracking with deep learning and geometry constraints. Proceedings of the IEEE International Conference on Computer Vision (ICCV), IEEE.

[49] Wang Z., Zhao J., Lu C., Huang H., Yang F., Li L., Guo Y. Learning to detect head movement in unconstrained remote gaze estimation in the wild. Proceedings of the IEEE/CVF Winter Conference on Applications of Computer Vision (WACV), IEEE.

[50] Cheng Y., Feng L., Zhang X. (2018). Appearance-based gaze estimation via evaluation-guided asymmetric regression. Proceedings of the European Conference on Computer Vision (ECCV).

[51] Yu Y., Liu G., Odobez J.M. Improving few-shot user-specific gaze adaptation via gaze redirection synthesis. Proceedings of the IEEE/CVF Conference on Computer Vision and Pattern Recognition (CVPR), IEEE.

[52] Lindén E., Sjöstrand J., Proutiere A. Learning to personalize in appearance-based gaze tracking. Proceedings of the IEEE/CVF International Conference on Computer Vision (ICCV), IEEE.

[53] Guo Z., Yuan Z., Zhang C., Chi W., Ling Y., Zhang S. Domain adaptation gaze estimation by embedding with prediction consistency. Proceedings of the Asian Conference on Computer Vision (ACCV).

[54] Yu Y., Odobez J.M. Unsupervised representation learning for gaze estimation. Proceedings of the 2020 IEEE/CVF Conference on Computer Vision and Pattern Recognition (CVPR), IEEE.

[55] Dubey N., Ghosh S., Dhall A. Unsupervised learning of eye gaze representation from the web. Proceedings of the 2019 International Joint Conference on Neural Networks (IJCNN), IEEE.

[56] Tonsen M., Zhang X., Sugano Y., Bulling A. Labelled Pupils in the Wild: A Dataset for Studying Pupil Detection in Unconstrained Environments. Proceedings of the Ninth Biennial ACM Symposium on Eye Tracking Research & Applications.

[57] Fuhl W., Kübler T., Sippel K., Rosenstiel W., Kasneci E. EXCUSE: Robust Pupil Detection in Real-World Scenarios. Proceedings of the Computer Analysis of Images and Patterns: 16th International Conference.

